# Situational Context and Perceived Threat Modulate Approachability Judgements to Emotional Faces

**DOI:** 10.1371/journal.pone.0131472

**Published:** 2015-06-29

**Authors:** Megan L. Willis, Natalie A. Windsor, Danielle L. Lawson, Nicole J. Ridley

**Affiliations:** 1 School of Psychology, Australian Catholic University (ACU), Sydney, New South Wales, Australia; 2 ARC Centre of Excellence in Cognition and its Disorders, School of Psychology, Australian Catholic University, Sydney, New South Wales, Australia; 3 School of Psychological Sciences, Australian College of Applied Psychology (ACAP), Sydney, New South Wales, Australia; University of Tuebingen Medical School, GERMANY

## Abstract

Facial expressions of emotion play a key role in guiding social judgements, including deciding whether or not to approach another person. However, no research has examined how situational context modulates approachability judgements assigned to emotional faces, or the relationship between perceived threat and approachability judgements. Fifty-two participants provided approachability judgements to angry, disgusted, fearful, happy, neutral, and sad faces across three situational contexts: no context, when giving help, and when receiving help. Participants also rated the emotional faces for level of perceived threat and labelled the facial expressions. Results indicated that context modulated approachability judgements to faces depicting negative emotions. Specifically, faces depicting distress-related emotions (i.e., sadness and fear) were considered more approachable in the giving help context than both the receiving help and neutral context. Furthermore, higher ratings of threat were associated with the assessment of angry, happy and neutral faces as less approachable. These findings are the first to demonstrate the significant role that context plays in the evaluation of an individual’s approachability and illustrate the important relationship between perceived threat and the evaluation of approachability.

## Introduction

In everyday life, we constantly make inferences about the characteristics of others on the basis of their facial appearance. This assessment can influence small decisions about our behaviour, such as who we decide to sit next to on a bus, to choices with more significant ramifications, such as who we decide to help or ask out on a date [1]. One social judgement that has been of particular interest to researchers in recent years is approachability, which refers to the likelihood of moving towards another individual (e.g., [2,3–5]). This judgement is particularly important for successful social navigation, given that deficits in the ability to accurately judge the appropriateness of engaging with another individual could have dramatic ramifications on an individual’s wellbeing [6]. Indeed, abnormalities in these social judgements can result in socially inappropriate, and in extreme cases, risky behaviour. Such abnormalities have been observed in individuals within several clinical populations, including people with bilateral amygdala lesions, autism spectrum disorder, schizophrenia, and Williams syndrome [7–10]. In contrast, healthy adults are highly consistent in how they make approachability judgements, which suggests that we rely on similar cues when making these judgements [7,11]. Angry faces and disgusted faces tend to be considered the least approachable, followed by fearful and sad faces (moderately unapproachable), with neutral and happy faces, in turn, considered the most approachable (e.g., [2,4]).

The divergent influence that discrete facial expressions exert on approachability judgements illustrates their capacity to influence social behaviour in distinct ways. But what accounts for the nature of these effects? Perceived threat is one factor that is thought to be influential in how approachability judgements are made. The amygdala appears to play an important role in social judgements [7,12] and is thought to be part of a neural system responsive to potential threat [2,12]. In addition, expressions that signal direct threat are also those deemed the least approachable by healthy adults [2]. It is widely acknowledged that facial expressions serve a communicative function that can influence the behaviour of observers (e.g., [13,14]). For instance, happiness is thought to communicate a lack of threat, and signal a desire to cooperate [15], while sadness reflects a desire to appease and may be used to elicit sympathy from the social group [16]. Anger and fear alert of potential threat, however differ in the meaning assigned to this threat [17]. Fearful faces are thought to signal the presence of a significant source of danger in the environment, which may signal to an onlooker to be vigilant for danger [18,19]. Anger, alternatively, signals a more direct and immediate threat from the expresser, particularly when aggression is directed at the observer [20]. The relevance of threat to approachability ratings is supported by findings that threatening faces (in particular, angry faces) are rated as less approachable than other negative expressions (e.g., sadness), positive expressions (e.g., happiness) and non-expressive neutral faces [4,21]. Interestingly, disgusted faces are rated as unapproachable as angry faces [2]. An explanation for this within the threat perception framework is that disgust represents a form of direct threat–elicited in response to socio-moral violations–as well as indirect threat–such as in contamination disgust, which reflects a concern with interpersonally transmitted disease (e.g., sharing a drink bottle; [22]). It may be that when making approachability judgements, disgusted faces are perceived to be as threatening as angry faces because they are perceived to be conveying direct threat. However, empirical evidence of an association between threat ratings and approachability judgements to emotional faces is yet to be demonstrated.

The evidence to date provides us with an understanding of how approachability judgements are influenced by facial expressions (e.g., negatively valenced expressions rated less approachable than positive) and points to the likely contribution of threat evaluation when making these judgements. However, this only provides us with a partial understanding of how these social judgements are made in daily life. Facial expressions are typically encountered in the context of a social situation, and it is well documented that context can modulate the speed and accuracy of facial expression recognition [23–31]. It is however, not clear how context affects the way we assign social judgements to emotional faces–a skill which is understood to be functionally dissociable from the capacity to recognise facial expressions [32,33].

The particular context in which an expression is encountered may play a significant role in what message is conveyed by a facial expression and the response of the observer to that expression, such as situational factors that may signal danger, or the relative power balance of the dyad [34]. For instance, fear observed in a context where danger is apparent (e.g., fleeing from a fire) may facilitate ‘flight’ behaviours, but in other contexts encourage prosocial behaviour by indicating that the expresser is helpless and in distress [35]. This point was illustrated by Marsh et al. [17], who found that despite presumptions that the fear expression is a predominantly aversive signal, fear also facilitates approach behaviours in perceivers. This is consistent with the suggestion that one function of the fear facial expression may be to elicit prosocial behaviour from perceivers [35]. Context may also have an influence on tendencies to approach or avoid in relation to the power dynamics of the dyad [36]. It has been hypothesised that elevated power may increase the tendency to perceive rewards and opportunities in ambiguous acts and interactions, encouraging approach, while reduced power may result in the individual interpreting ambiguous events as more threatening [36]. This perspective would suggest that individuals are more likely to approach others if they have power (e.g., have the option to provide help or information to others) and are more vigilant to markers of threat if they lack power (e.g., need assistance). However, this proposed influence of context–including differences in threat and power—on approachability judgements has not yet been assessed.

To date, approachability tasks have assessed judgements without a specific context, (e.g., ‘How approachable is this person?’) or in a receiving help context (e.g., where the judger needs to approach someone for help) [2,4,5,21,32]. To our knowledge, no study has attempted to investigate approachability in a ‘giving help’ context; that is, how likely one would be to approach the person if the context suggests that the person being evaluated is in need of help. In approachability research to date, faces depicting the distress-related emotions of fear and sadness have also been judged as unapproachable, albeit to a lesser extent than angry and disgust faces [2]. However, as mentioned previously, fear and other distress-related emotions, like sadness, are thought to serve the adaptive purpose of eliciting prosocial behaviours; eliciting not only the desire for affiliation but caregiving from the social group [35,37,38]. It is possible that context mediates the precise approachability judgement assigned to emotional faces. For instance, when an individual needs help they may perceive individuals expressing sadness or fear as unapproachable, but when the context suggests the individual being evaluated is in need of help they might perceive individuals expressing these same emotions as more approachable. This hypothesis is plausible in view of both the hypothesised influence of power on approachability judgements as well as the adaptive nature of facial expressions. In addition, despite the accumulation of evidence that has pointed to a potential role of threat in how these judgments are made, no study to date has directly examined the relationship between perceived threat and the precise approachability judgements assigned to emotional faces.

The first aim of the current study was to examine how context affects one’s willingness to approach individuals depicting various facial expressions of emotion. The second aim was to examine the relationship between threat and approachability judgements given its purported relevance to these judgements. We contrasted approachability judgements assigned to emotional faces (i.e., angry, disgusted, fearful, happy, neutral and sad) across three contexts–when no contextual information was provided, when considering approaching individuals to receive help, and considering approaching to give help. We anticipated that faces displaying the distress-related emotions of sadness and fear would be considered more approachable in the giving help context than the receiving help context and neutral context. We also collected threat ratings assigned to faces of each emotional category. This enabled us to empirically test the hypothesis that the nature of approachability judgements assigned to emotional faces is associated with the level of threat perceived in the face. We anticipated that threat perception ratings would inversely mirror social judgement ratings (e.g., the expressions rated the least approachable, such as angry and disgusted faces, would be perceived as most threatening), with enhanced threat ratings associated with the evaluation of faces as less approachable for all emotions. We also measured facial expression recognition accuracy to confirm accurate categorisation of emotions by participants.

## Method

### Ethics Statement

This research was approved by the Australian Catholic University’s Human Research Ethics Committee (HREC). All participants provided written informed consent to participate in the study.

### Participants

Fifty-two (39 female) individuals participated in the experiment in return for course credit or entry into a prize draw. Ages ranged from 18 to 53 (*M* = 23.75, *SD* = 7.68). All participants had normal or corrected-to-normal vision and no history of brain injury.

### Stimuli

Faces of 10 individuals (five male) were sourced from the Karolinska Directed Emotional Faces (KDEF) database [39]. Photographs of each individual displaying an angry, disgusted, happy, sad, fearful and neutral pose were selected, for a total of 60 faces. The faces (256 grey levels, 72 ppi) were scaled to be of equivalent size and displayed within a black rectangular background of 6.3 cm x 8.5 cm, which subtended a visual angle of approximately 6.01° by 8.10° at the experimental resolution.

### Approachability Tasks

#### No context

In this task, participants judged the approachability of the aforementioned 60 faces. They were not given any contextual information upon which to base their judgement. For each face, they were instructed to indicate their agreement with the statement “I would approach this person”. The faces were shown one at a time on a white background, in a randomised order. Participants indicated the extent of their agreement with the statement on a 9-point Likert scale ranging from -4 (strongly disagree) to +4 (strongly agree). The stimulus was presented in the middle of the screen with the statement presented above the face and the response scale presented below the face. The stimulus, scale and statement remained on the screen until a response was made by way of mouse click. An inter-trial interval of 500ms preceded the onset of the next trial.

#### Receiving help

Participants completed the ‘receiving help’ approachability task employed in previous research [2,4,5,32]. In this task, participants were instructed to imagine being on a crowded street on their way to meet a friend. They were asked to pretend that they were lost and in a hurry and needed to ask someone for directions. They were then requested to imagine seeing each person in the crowd and to indicate the extent to which they agreed with the following statement “I would approach this person to ask for directions.” All other aspects of the task were identical to that described for the no context task.

#### Giving help

Participants were instructed to imagine leaving their local library and seeing a person carrying a pile of books trip and drop the books. For each person, participants were asked to indicate the extent to which they agreed with the following statement “I would approach this person and offer them help”. All other aspects of the task were identical to the other two approachability tasks.

### Threat Perception Task

In this task, participants were asked to rate how threatening they found each face. Responses were made on a 9-point Likert scale from 0 (not at all threatening) to 8 (extremely threatening). The response scale was presented underneath each image. The presentation of stimuli, method of response and inter-trial interval, were as described for the approachability tasks.

### Facial Expression Recognition

Participants also completed a facial expression recognition task. Participants were shown each face and were asked to label each expression displayed as: angry, disgusted, fearful, happy, neutral or sad, by selecting the appropriate label from six options displayed below each face. Each image and the six emotion labels were displayed until a response was made. The presentation of stimuli, method of response and inter-trial interval, were as described for aforementioned tasks.

### Procedure

Each participant was tested individually in a quiet room. At the commencement of the study, participants provided demographic information. Following which, the three approachability tasks were completed. All participants completed the no context task first. This task was completed first in order to ensure that responses were not confounded with exposure to the other contexts. To determine if completing the no context condition first inflated the effect of contextual information on approachability judgements, we compared the data from the current study with unpublished data in which participants completed the giving help and receiving help contexts in a counterbalanced order, with no neutral context condition completed. There was no main effect of data set, nor any interaction with context and/or emotion. The giving help context and receiving help context tasks were then completed in a counterbalanced order between participants. There was no main effect of order, nor any interaction with context and/or emotion in the subsequent analyses. Participants then completed the threat perception task and finally, the facial expression recognition task. Stimulus presentation was controlled using Superlab (Cedrus Corp.) and viewed on a 13-inch monitor on a MacBook Pro Computer at a viewing distance of approximately 40 cm.

### Statistical Analyses

The primary analysis conducted was a two-way repeated measures analysis of variance (ANOVA) assessing mean approachability ratings for the repeated measures factors of context (no context, giving help and receiving help) and emotion (angry, disgusted, fearful, happy, neutral and sad). Mean threat perception ratings and mean emotion recognition accuracy were analysed using one-way repeated measures ANOVAs with the repeated measures factor of emotion (angry, disgusted, fearful, happy, neutral and sad). The Greenhouse-Geisser epsilon adjusted value is reported in all instances where the sphericity assumption was violated. Given the large sample size (e.g., n > 30), the sample was assumed to come from a normal sampling distribution [40]. Finally, Pearson’s correlations were performed between threat perception ratings and approachability ratings in each context. The complete data spreadsheet can be found in S1 Data.

## Results

### Approachability

**Fig 1:**
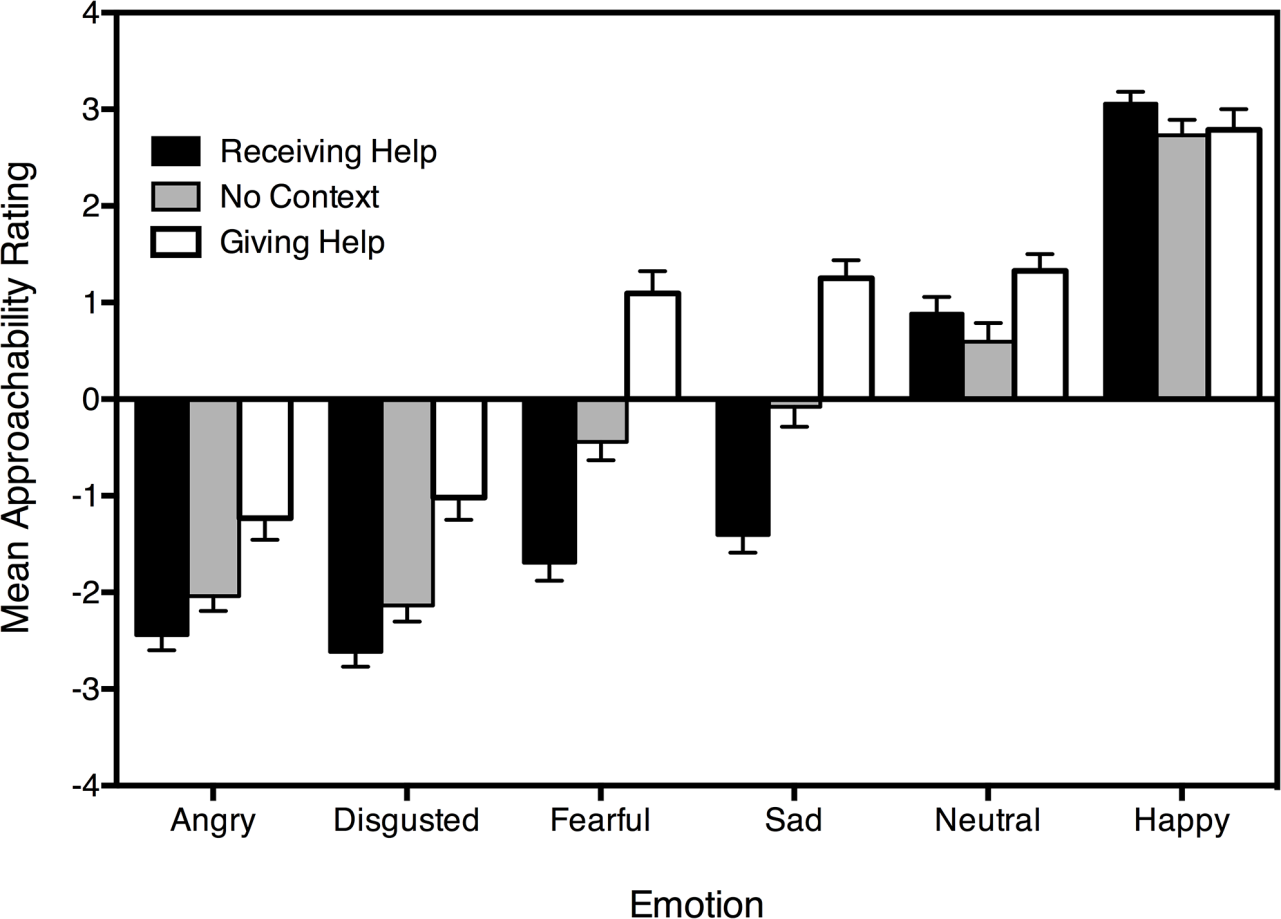
Mean approachability ratings for faces of each expression across the three contexts. Standard error bars are shown.

Mean approachability ratings assigned to emotional faces across the three contexts are displayed (Fig 1). Results revealed significant main effects of context, *F*(2, 102) = 71.39, *p* < .001, η_p_^2^ = .58, and emotion, *F*(3.13, 159.83) = 299.70, *p* < .001, η_p_^2^ = .85, which were moderated by a significant Context × Emotion interaction, *F*(5.64, 287.41) = 32.59, *p* < .001, η_p_^2^ = .39, indicating that the influence of emotional expression on approachability ratings was modulated by context.

Simple main effects analyses (Bonferroni adjusted) were performed to investigate the significant interaction that emerged. We compared approachability ratings between the three contexts, separately for each emotion. The *t*, *p* and Cohen’s *d* values for these comparisons are presented in Table 1. Angry, disgusted, fearful, sad and neutral faces were rated as significantly more approachable in the giving help context than both the receiving help context and when there was no context. Angry, disgusted, fearful and sad faces were judged as significantly less approachable in the receiving help context than when there was no context. In contrast, there was no significant difference between approachability judgements assigned to neutral faces in the receiving help context, compared to when there was no context. There was no significant difference between approachability judgements assigned to happy faces across the three contexts.

**Table 1:** Inferential statistics for paired-sample t-tests comparing approachability ratings between contexts, separately for each emotion.

	Context								
	Giving Help vs No Context			Giving Help vs Receiving Help			Receiving Help vs No Context		
Emotion	*t*(51)	*p*	*d*	*t*(51)	*p*	*d*	*t*(51)	*p*	*d*
Angry	4.16	.002	0.56	6.04	< .001	0.84	3.65	.011	0.36
Disgusted	6.22	< .001	0.74	7.89	< .001	1.09	3.78	.008	0.41
Fearful	7.59	< .001	1.00	11.78	< .001	1.83	6.51	< .001	0.92
Happy	0.29	1.00	0.04	1.59	1.00	0.20	2.68	.178	0.31
Neutral	4.62	< .001	0.55	3.26	.036	0.35	1.97	.970	0.22
Sad	6.85	< .001	0.92	11.64	< .001	1.97	6.01	< .001	0.93

One-sample t-tests (Bonferroni adjusted) were performed comparing mean approachability ratings to the neutral approachability value of 0. The *t*, *p* and Cohen’s *d* values for these comparisons are presented in Table 2. In all contexts, angry and disgusted faces were considered unapproachable. In contrast, happy faces were considered approachable in all contexts. As anticipated, judgements assigned to neutral, sad and fearful faces differed across the three contexts. Whereas sad and fearful faces were judged unapproachable in the receiving help context, they were considered approachable in the giving help context, and ratings did not differ significantly from the neutral point of 0 when there was no context. Finally, neutral faces were judged approachable in the giving and receiving help contexts. When there was no context, the trend for neutral faces to be judged approachable failed to reach significance after adjustments for multiple comparisons.

**Table 2:** Inferential statistics for one-sample t-tests comparing approachability ratings in each context to the neutral value of zero, separately for each emotion.

	Context								
	Giving Help			No Context			Receiving Help		
Emotion	*t*(51)	*p*	*d*	*t*(51)	*p*	*d*	*t*(51)	*p*	*d*
Angry	5.49	< .001	0.76	13.13	< .001	1.82	15.62	< .001	2.17
Disgusted	4.42	< .001	0.61	12.77	< .001	1.77	17.06	< .001	2.37
Fearful	4.76	< .001	0.66	2.32	.444	0.32	9.03	< .001	1.25
Happy	13.07	< .001	1.81	17.03	< .001	2.36	24.64	< .001	3.42
Neutral	7.67	< .001	1.07	3.13	.053	0.44	5.12	< .001	0.71
Sad	6.65	< .001	0.92	0.37	1.00	0.05	7.60	< .001	1.05

### Threat Perception

**Fig 2:**
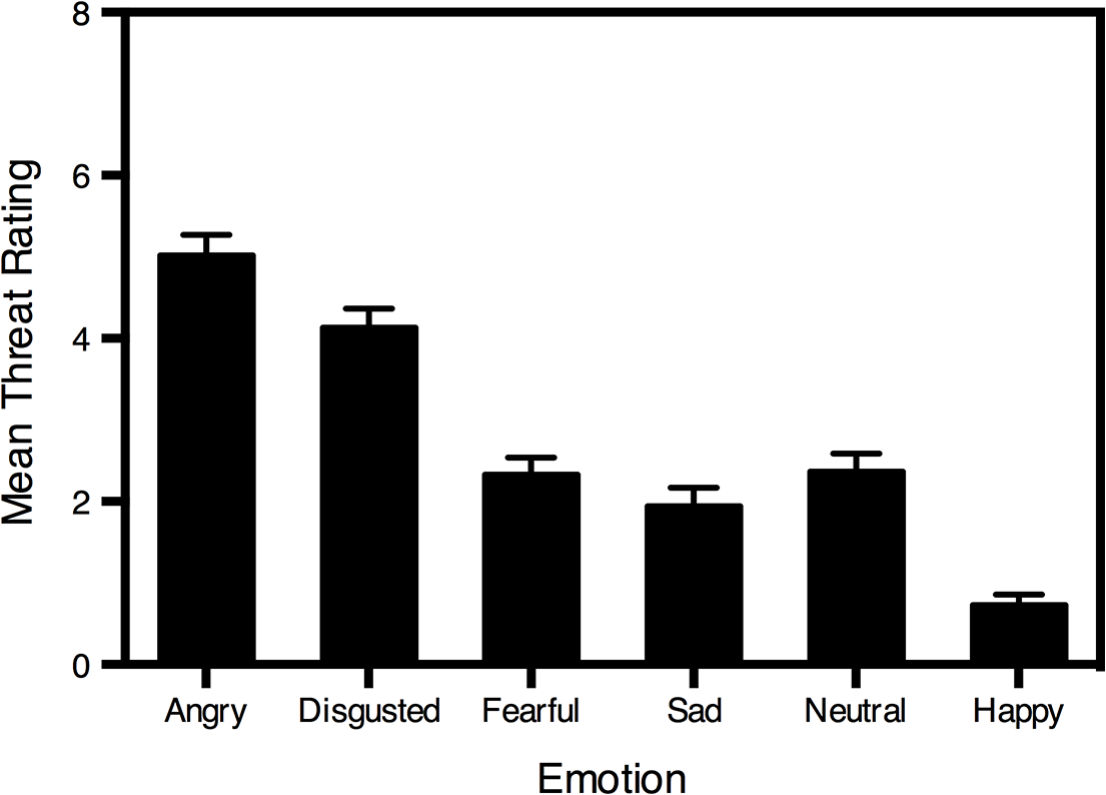
Mean threat ratings for faces of each expression. Standard error bars are shown.

Mean threat ratings for faces of each expression are displayed (Fig 2). Results revealed a significant main effect of emotion, *F*(2.67, 136.12) = 123.61, *p* < .001, η_p_^2^ = .71.

The *t*, *p* and Cohen’s *d* values for follow up pairwise comparisons (Bonferroni adjusted) are presented in Table 3. Angry faces were perceived as significantly more threatening than all other facial expressions, and disgusted faces were rated as significantly more threatening than fearful, sad, neutral and happy faces. Fearful faces were considered significantly more threatening than sad and happy faces. Similarly, sad and neutral faces were perceived as significantly more threatening than happy faces. Threat ratings assigned to neutral faces did not differ significantly from those assigned to fearful and sad faces.

**Table 3:** Inferential statistics for paired-sample t-tests comparing threat perception ratings between. emotions.

Comparison	*t*(51)	*p*	*d*
Angry vs Disgusted	7.27	< .001	0.50
Angry vs Fearful	12.60	< .001	1.59
Angry vs Happy	16.81	< .001	2.90
Angry vs Neutral	11.32	< .001	1.54
Angry vs Sad	11.39	< .001	1.78
Disgusted vs Fearful	10.40	< .001	1.13
Disgusted vs Happy	14.89	< .001	2.47
Disgusted vs Neutral	8.14	< .001	1.09
Disgusted vs Sad	10.06	< .001	1.34
Fearful vs Happy	9.05	< .001	1.19
Fearful vs Neutral	0.23	1.00	0.02
Fearful vs Sad	3.60	.011	0.24
Happy vs Neutral	9.92	< .001	1.11
Happy vs Sad	7.03	< .001	0.81
Neutral vs Sad	2.40	.304	0.26

### Facial Expression Recognition

**Fig 3:**
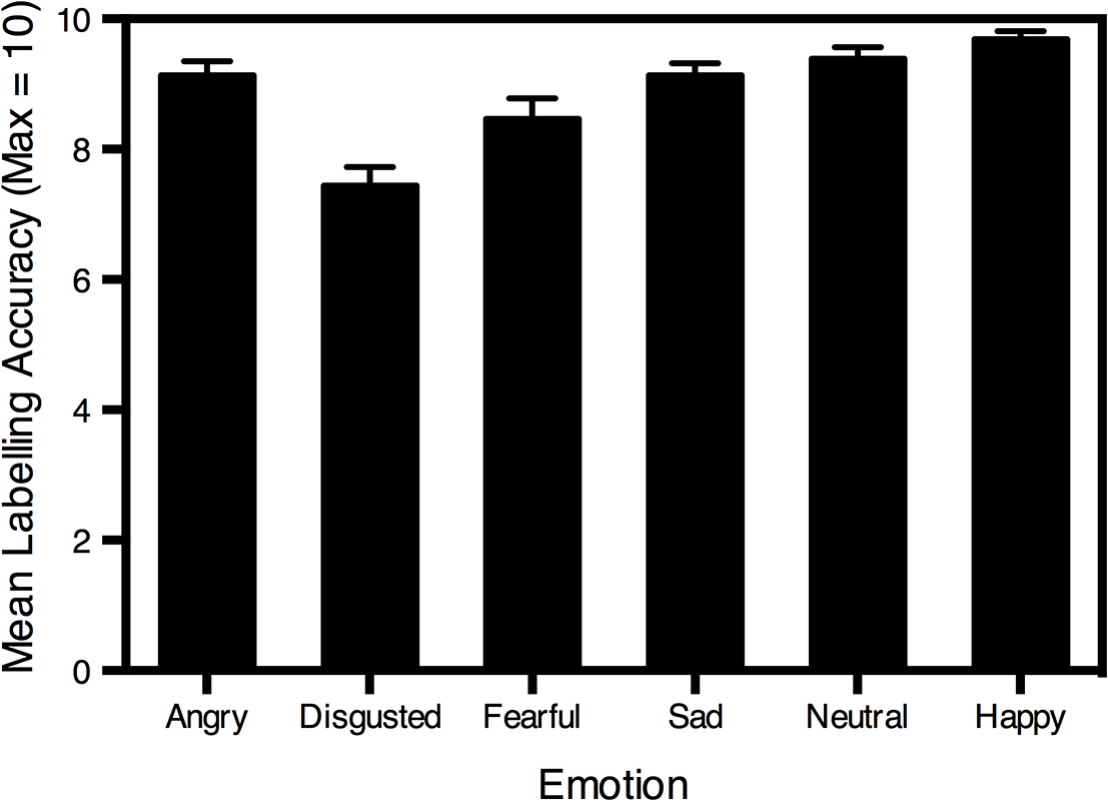
Mean emotion labelling accuracy for faces of each expression. Standard error bars are shown.

Mean facial expression recognition accuracy for faces are displayed (Fig 3). Analysis of facial expression recognition data revealed a significant main effect of emotion, *F*(3.66, 186.67) = 15.58, *p* < .001, η_p_^2^ = .23. The *t*, *p* and Cohen’s *d* values for follow up pairwise comparisons (Bonferroni adjusted) are presented in Table 4. Happy facial expressions were recognised most accurately, significantly more so than disgusted and fearful faces. Disgusted facial expressions were recognised significantly less accurately than angry, neutral and sad facial expressions. No other significant differences emerged for all remaining comparisons.

**Table 4:** Inferential statistics for paired-sample t-tests comparing facial expression recognition accuracy between emotions.

Comparison	*t*(51)	*p*	*d*
Angry vs Disgusted	5.20	< .001	0.92
Angry vs Fearful	1.93	.881	0.32
Angry vs Happy	2.68	.148	0.43
Angry vs Neutral	0.87	1.00	0.17
Angry vs Sad	0.00	1.00	0.00
Disgusted vs Fearful	2.51	.232	0.47
Disgusted vs Happy	7.89	< .001	1.37
Disgusted vs Neutral	6.12	< .001	1.11
Disgusted vs Sad	5.86	< .001	0.95
Fearful vs Happy	3.98	.003	0.66
Fearful vs Neutral	2.80	.107	0.48
Fearful vs Sad	2.21	.472	0.34
Happy vs Neutral	1.70	1.00	0.27
Happy vs Sad	2.61	.177	0.49
Neutral vs Sad	1.03	1.00	0.19

### Correlating Approachability Ratings and Threat Perception

Table 5 displays Pearson correlations between perceived threat ratings for each expression and approachability ratings assigned to each expression, separately for each context. As Table 1 illustrates, across the three contexts, greater perceived threat ratings were significantly associated with more negative approachability ratings to angry, happy and neutral faces. The perception of threat was only significantly associated with approachability ratings assigned to sad and fearful faces when there was no contextual information provided, with more negative approachability ratings associated with heightened threat perception. While there was no significant relationship between threat and approachability ratings assigned to disgusted faces when there was no context, significant negative correlations emerged in the giving help and receiving help contexts, such that more threatening ratings of disgusted faces were associated with more negative approachability ratings.

**Table 5:** Correlations between threat ratings and approachability judgements to emotional faces, separately for each context.

	Context		
Emotion	Giving Help	No Context	Receiving Help
Angry	– .37**	– .40**	– .50**
Disgusted	– .31*	– .23	– .33*
Fearful	– .27	– .35*	– .16
Sad	– .20	– .39**	– .15
Neutral	– .55***	– .45***	– .52***
Happy	– .36**	– .39**	– .49***

* *p* < .05

** *p* < .01

*** *p* < .001

## Discussion

The current study sought to address two primary aims. First, we investigated whether context influenced the perceived approachability of angry, disgusted, fearful, happy, neutral, and sad faces. Second, we examined whether threat ratings assigned to the aforementioned expressions were associated with approachability judgements assigned to the same faces. The central finding of our study is that context modulated approachability judgements to faces depicting negative emotions. While the influence of context on perception of facial expressions in the facial expression recognition literature has been well documented [24], this study is the first to demonstrate that context modulates how facial expressions influence judgements of approachability.

As anticipated, faces depicting distress-related emotions (i.e., sadness and fear) were considered more approachable in the giving help context than in the receiving help and no context conditions. In addition to angry, disgusted, fearful and sad faces, neutral faces were considered significantly more approachable in the giving help context compared to when there was no context. However, approachability judgements assigned to neutral faces in the receiving help context did not differ significantly from those assigned to neutral faces in the giving help context and when there was no contextual information provided. Finally, for happy faces, context did not modulate the nature of approachability judgements assigned. These findings suggest that context is most critical in influencing approachability judgements for negative emotions, most likely because there are more obvious potential negative ramifications associated with approaching an individual expressing a negative emotion (i.e., threat). Moreover, context appears to play a fundamental role in the extent to which distress can evoke assistance, as distress-related faces were considered more approachable in an appropriate context. This is consistent with research on the bystander intervention which has shown that assistance is more likely when the cause of distress in unambiguous [41].

Our second key finding was, as anticipated, that explicit threat ratings were associated with approachability ratings for angry, happy and neutral faces, regardless of context. That is, heightened perception of threat was associated with the evaluation of these emotional faces as less approachable. Threat ratings for disgusted, fearful and sad faces were also negatively correlated with approachability ratings, however these relationships did not reach statistical significance in all contexts. Specifically, heightened perception of threat was associated with the evaluation of fearful and sad faces as less approachable when there was no context, and for disgusted faces in the giving and receiving help contexts, but not when there was no context. As expected, when threat ratings were viewed in isolation, ratings of threat varied according to directness of threat conveyed–angry faces were perceived as significantly more threatening than all other facial expressions, followed by disgusted faces, while fearful faces were rated as more threatening than sad and happy faces, but not neutral faces. Our proposal that approachability ratings are modulated by threat is supported by the finding that faces that signalled direct threat (i.e., angry and disgusted) were considered significantly more approachable in the giving help context than the receiving help context and when there was no context. This suggests that a perceiver may be more inclined to approach an individual displaying these emotions when the cause is apparent and suggests that harm is unlikely to be directed towards them. In addition, these findings are consistent with the hypothesis that increased power (e.g., the freedom to provide resources) is related to increased approach-related behaviours, while reduced power is associated with increased vigilance to threat and a reduced tendency to approach [36]. It is notable that angry, happy and neutral expressions are typically associated with ‘approach behaviours’, whilst sad, fearful and disgusted expressions are thought to represent more ‘avoidance oriented’ intentions [42]. The significant relationship of ratings of threat to approachability judgements across contexts for approach-oriented expressions may reflect the vigilance that is necessary for expressions that typically suggest confrontation, while observation of an individual with avoidance-oriented intentions may be less influenced by the threat expressed by that facial expression, given that individual may be less likely to approach. It is clear that one limitation in our ability to generalise the influence of perceived threat to approachability ratings in relation to contexts was that threat ratings were made in one context only–where no context was provided. While the aim of this current study was to examine the relationship between threat ratings and approachability, rather than to investigate the relationship of threat and context, obtaining threat ratings from participants in all three contexts could allow further examination of whether perceived threat is modulated by context, and its relationship to approachability ratings. In addition, replication of this study with neuroimaging and behavioural measures in both healthy adult and clinical populations may help to delineate what brain structures are pivotal in the assessment of threat evaluation and approachability, and any overlap in the neural correlates of these judgements. For instance, neuroimaging studies have demonstrated that healthy individuals display increased amygdala activation when presented with those faces that are ascribed the most negative social judgements [43], and individuals with evidence of abnormal amygdala functioning, such as those with bilateral amygdala lesions, schizophrenia and Williams syndrome–demonstrate abnormal judgements when judging whether to approach others [7,9,10,44]. Replication of this study in conjunction with functional neuroimaging in both healthy adults and clinical populations would help clarify the extent to which the function of the amygdala and other brain structures linked to social decision-making (e.g., the orbitofrontal cortex [32]) are pivotal to judgements of approachability and threat, and the behavioural implications of any impairment to these brain structures.

Interestingly, while the effect of context was similar for angry and disgusted faces, a difference did emerge on the explicit threat-rating task–which was not conducted by Willis et al. [2] study–angry expressions were rated as significantly more threatening than disgusted expressions. Willis et al. [2] found that disgusted faces were rated highest in emotional intensity; it is possible that negative social judgements assigned to disgusted faces relate to the perception of emotional intensity in combination with the perception of threat, that is, both emotional intensity and perceived threat have additive effects when it comes to determining how approachable disgusted faces are. Given that disgust is an ambivalent expression that can signal either direct or indirect threat [22], future research could examine what circumstances dictate how the disgust expression is perceived, the effect of intensity and how these factors in combination influence social judgements.

A number of investigations could build upon our findings. Given that correlations between threat and approachability ratings did not account for the full variance in approachability judgements, further research is required to ascertain what other factors influence how these judgements are made. Individual differences such as trait anxiety and levels of empathy, for instance, have been shown to mediate social judgements [5,38]. Age may also influence how these judgements are made; Slessor et al. [3] found that older (65+) adults were more likely to approach individuals who directly expressed anger, compared to younger adults. Investigation of what precise individual characteristics influence approachability judgements, and in what contexts these individual differences are most influential, would provide a more accurate picture of ‘real-life’ social decision-making. In addition, other individual differences such as culture [45], sex [34] have been shown to influence assessment and attribution of facial expressions. The purpose of the current research was not to explore these influences, however these are sources of variability may modulate how approachability judgements are made, and warrant consideration in future research on approachability judgements.

Manipulation of situational variables, also, would provide us with further insights into the relationship of perceived threat and approachability judgements. In our contextual example, we presented a scenario where the facial expression was interpreted as a reaction to an inconvenience–the expresser dropping books–and any real threat to the observer was minimal. Some facial expressions could be seen as more congruent to this context than others (e.g., anger would be more appropriate than disgust), and given the documented influence of congruency of the contextual scene on perception of facial expressions in facial expression recognition paradigms (for a review, see [24]), the extent of congruency may have had an influence on approachability judgements. Replication of this study with a broader range of scenarios would help to determine how results vary with differing causes of the expressed emotion, which vary in their congruency to the context. While the results of this current study suggest that evaluation of negative expressions in particular are most sensitive to the effects of context, other authors have demonstrated that the presence of threatening cues, whether evident in the face, body or surrounding scene, specifically directs the visual attention of the observer in a manner that renders the effects of peripheral contextual variables as less influential [23,46]. Further manipulation of contextual variables–such as providing an ‘avoidance’ oriented scenario to complement the ‘approachability’ focus of this current study–may help to clarify whether the stable positive evaluations to happy faces is a constant finding across situational contexts. In addition, it would be interesting to examine the likelihood of prosocial behaviour if the level of threat within the scenario was manipulated. Marsh and Ambady [35] suggest that in a situation in which some danger is apparent, fear would be interpreted as a threat cue rather than eliciting approach responses (e.g., a building smelling of smoke). Further research could examine the approach/avoid behaviours to facial expressions as a function of situational factors (particularly potential harm), further extending our understanding of contextual influences on social responses.

In conclusion, we have demonstrated that context influences the perceived approachability of individuals displaying negatively valenced facial expressions. Importantly, individuals displaying distress-related emotions of fear and sadness are considered more approachable when the context suggests they are in need of help, despite having lower approachability ratings in the context of giving help and when no contextual information is provided. All negatively valenced facial expressions were viewed as more approachable in the giving help context than in the receiving help context and when no contextual information was provided. Our results also support the assumed critical role of perceived threat in judgements of approachability. Expressions thought to represent direct forms of threat (i.e., anger and disgust) were rated less approachable than other expressions that represented indirect or minimal forms of threat, regardless of context. Heightened threat ratings corresponded with negative approachability ratings for angry, happy and neutral expressions for all contexts, suggesting vigilance to threat is a constant influence for these ‘approach-related emotions’. It is clear that further studies investigating the relationship between facial expressions and approachability need to account for the impact of context if a full model of social behaviour is to be established. Collectively, these results provide support for the suggestion that facial expressions have evolved because of their adaptive capacity to influence an onlooker’s social behaviour, including whether it is adaptive to approach or avoid the expresser [47]. These findings also reveal that our understanding of the social function of facial expressions is inextricably linked to social context and illustrates the importance of considering context in our pursuit to understand their function in our social interactions.

## Supporting Information

S1 Data S1 Data File provides all data on which reported analyses were performed. (XLSX)
